# The role of homeobox genes in signaling pathway positive feedback loops and cancer therapy

**DOI:** 10.3389/fonc.2026.1881148

**Published:** 2026-06-22

**Authors:** Ben Zion Vider

**Affiliations:** Independent Researcher, Moshav Herut, Israel

**Keywords:** epigenetic drugs, homeobox genes, positive feedback loop, RAS inhibitors, RTK inhibitors, signaling pathway, tumor plasticity

## Abstract

In this Perspective, the interaction between receptor components of signaling pathways and downstream transcription factors, particularly homeobox genes, is examined. The manuscript proposes that positive-feedback loops between pathway receptor components and downstream transcription factors represent a central organizing principle in development, normal cellular function, and tumor plasticity. This aspect of signaling pathway function is often overlooked, and its implications for cancer progression and therapy remain insufficiently explored. Various possible forms of this mechanism are presented. The biological logic behind this mechanism is discussed. Several prominent examples of its operation are suggested, particularly in relation to receptor tyrosine kinase (RTK)–RAS-related signaling pathways and their homeobox gene outputs in both normal and cancer states. Special attention is devoted to epithelial–mesenchymal transition (EMT), a process central to many cancers. Based on this concept, the manuscript briefly outlines the potential use of RTK- or RAS-targeted inhibitors together with epigenetic drugs in cancer therapy, with the goal of simultaneously blocking both the input and the transcriptional output of mutation-activated signaling pathways.

## Introduction

Signals that begin at cell-surface receptors and end in the activation of transcription factors are central components of cell function. They form signaling pathways that regulate development, cellular differentiation, normal cellular function, and tumor plasticity. According to this Perspective, one of the central principles by which these pathways function is the formation of positive-feedback loops, in which transcription factors activated within a signaling pathway direct the transcription of genes that belong to the same pathway, thereby stabilizing pathway activity within the cell (1). This aspect of signaling pathway function is often overlooked, and its implications for cancer progression and therapy therefore remain insufficiently explored.

In cancer, hyperactivation of signaling pathways due to mutations in genes such as receptor tyrosine kinases (RTKs) and RAS plays a central role in disease progression. These pathways include the RAS-driven RAF–MEK–extracellular signal-regulated kinase (ERK) and phosphoinositide 3-kinase (PI3K)–AKT–mechanistic target of rapamycin (mTOR) branches, hereafter referred to as the RTK–RAS signaling network (2, 3).

Homeobox transcription factors act as central downstream effectors of this signaling network, and each of them may regulate hundreds of target genes, both in normal and cancer states (4–9). In this manner, homeobox transcription factors are positioned not only as downstream effectors of signaling pathways, but also as possible regulators of upstream signaling components. When these upstream components belong to the same signaling pathway, they may enable the formation of self-reinforcing regulatory circuits.

The function of the RTK–RAS signaling network acting through homeobox genes is examined here through the concept of positive feedback in several processes, both in normal cells and in cancer cells. Epithelial–mesenchymal transition (EMT) is one central example (10). In addition, a particularly clear example of another positive-feedback loop is described in breast cancer, and the conclusions drawn from it are also discussed (11).

Based on these concepts, this Perspective outlines the combined use of RTK- or RAS-targeted inhibitors together with epigenetic drugs in cancer therapy, with the goal of blocking the transcriptional output of mutation-activated signaling pathways, together with the resulting tumor-progression properties and resistance to therapy.

It is important to note that some of the positive-feedback loops described here are inferred from the literature by joining two parts of a loop into a complete functional circuit. In this way, the “detective” work involved in identifying these loops becomes an invitation to prove them in further functional studies. However, some of the positive-feedback loops discussed in this Perspective have already been described as such in the works presented here.

## Cell signaling pathways

Cell signaling pathways are core functional components of cell biology. Various membrane proteins, many of them specific to different cell types, converge through several major pathways to transmit signals from the cellular environment into the nucleus. Upon reaching the nucleus, these signals activate cell-cycle responses and processes related to cell fate programming.

Among the most prominent of these pathways are those activated by RTKs (2). These receptors, expressed by most cells, simultaneously activate the ERK, PI3K–AKT, and stress mitogen-activated protein kinase (MAPK) pathways, including p38 and JNK, which interconnect at multiple junctions (3, 12).

The ERK signaling pathway activates several downstream transcription factors, including activator protein 1 (AP-1) family proteins such as FOS and JUN, E26 transformation-specific (ETS) transcription factors, MYC, and ELK1 (13). In parallel, the PI3K pathway primarily regulates the forkhead box O (FOXO) family and mTOR complex 1 (mTORC1)-dependent transcriptional programs, while also cooperating with transcription factors activated by ERK (14). Through these transcriptional outputs, ERK and PI3K signaling can regulate cell-type-specific members of the homeobox gene family, thereby linking upstream signaling activity to developmental and cancer-associated transcriptional programs (15, 16).

Once activated, these pathways also influence the epigenetic state of the genome by affecting DNA methylation and histone modifications, including changes in methylation, acetylation, and phosphorylation (17).

## Homeobox genes as executors of signaling-directed cellular programs

Homeobox genes are among the central families of transcription factors. They direct development, participate in cell-cycle regulation, and are deeply involved in cellular reprogramming in cancer (18, 19).

Homeobox transcription factors are positioned both as downstream effectors of signaling pathways and as regulators of upstream signaling components. When these upstream components belong to the same signaling pathway, they may enable the formation of self-reinforcing regulatory circuits (11).

In cancer, when signaling pathways are activated by oncogene activation or tumor-suppressor gene inactivation, their output becomes more robust and persistent. The transcription factors acting downstream of these pathways, including homeobox genes, may therefore become more strongly expressed or acquire altered patterns of expression.

In cancer, as in normal development, homeobox genes continue to execute cellular programs. The difference is that, in cancer, the program is no longer the normal developmental or tissue-maintenance program, with its strict regulation of the cell cycle and social cell behavior, but a malignant one. In this view, homeobox genes should not be regarded primarily as factors whose role is to preserve normal cellular balance. Rather, they are powerful executors of diverse cellular programs, whatever these programs may be.

## Logical assumptions underlying positive feedback in signaling pathways

Let us consider the function of a single cell that has just divided and begins to establish its identity. A new transcription factor, for example a homeobox gene, becomes activated by an intrinsic mechanism. This homeobox gene, through its protein product, directs the transcription of many genes, including components of a certain signaling pathway. If, while this signaling pathway functions, it does not further activate the homeobox gene responsible for its establishment, that homeobox gene may not remain active, as the intrinsic state of the cell is continuously changing. As a consequence, the signaling pathway components induced by that homeobox gene would no longer be transcriptionally supported. In such a case, the emerging cellular identity established by this homeobox gene would not become stabilized.

During development, however, cells can expand the spectrum of proteins they transcribe, including proteins that participate in signaling pathways that do not directly support that specific homeobox gene. This diversification is part of developmental progression. Nevertheless, the transcriptional program responsible for the current cellular identity must also be maintained; otherwise, that identity would not become stabilized.

A second issue relates to evolutionary persistence. Because genomes are subject to continuous change during evolution, transcription factors that become expressed must acquire functional significance for cellular fate. One way this may occur is through the promotion of cellular proliferation, thereby supporting the persistence of the transcriptional programs they regulate. This mechanism is plausible because signaling pathways frequently activate cell-cycle genes.

The participation of transcription factors, including homeobox genes, in signaling pathways results in two main outcomes. First, positive-feedback mechanisms reinforce signaling activity and intensify pathway output. Second, as master transcriptional regulators, homeobox genes expand the cellular protein repertoire in new directions. In cancer, this outcome is part of what we define as tumor plasticity or cancer cell reprogramming.

## Positive feedback in budding yeast mating as an early model of transcription factor-directed cellular programming

Several stations along evolution and in the cancerous state can be used to illustrate positive-feedback loops.

The central role of transcription factors in establishing cellular programs and positive-feedback loops is already evident in budding yeast mating (20). Mating is initiated when a cell-type-specific pheromone receptor is activated by pheromone secreted by the opposite mating type. This activation triggers a MAPK cascade that regulates the transcription factor Ste12, a protein with a homeodomain-like DNA-binding region showing 23% identity with Antp, including the highly conserved WFQNRR residues. Activated Ste12 induces mating-specific genes that promote cell-cycle arrest, agglutination, polarized growth, and conjugation. In this context, the induction of Ste12-dependent genes, including components of the pheromone-response pathway itself, such as pheromone receptors and signaling regulators, creates a feedback architecture that amplifies and stabilizes the mating program, including Ste12 expression itself.

## Upstream regulation of Hox gene expression and function

Relative to the number of studies that describe the targets of homeobox genes, far fewer studies describe the proteins that act on homeobox gene expression and function. One of these is the review by Primon et al. (4).

This review summarizes various events in which Hox proteins are phosphorylated by different kinases. Some of these kinases are components of signaling pathways, whereas others are related to the cell cycle or to other kinase activities in the cell. These phosphorylation events demonstrate diverse effects on Hox proteins, some of them cell-type specific and others related to the cell cycle.

Among the studies cited in this review, I would like to address one that presents a positive-feedback loop involving a signaling pathway and a Hox gene. This study, titled “JAK/STAT and Hox Dynamic Interactions in an Organogenetic Gene Cascade,” analyzes the induction of posterior spiracle organogenesis in Drosophila by the Hox gene Abdominal-B (Abd-B).

## JAK/STAT–Abd-B positive feedback during posterior spiracle organogenesis

In Drosophila, the induction of posterior spiracle organogenesis, which forms an air-pipe-like structure, is directed by the Hox gene Abdominal-B (Abd-B). Initially, Abd-B activates a cascade of transcription factors and signaling molecules in the spiracle primordium. These include the transcription factor genes cut (ct), empty spiracles (ems), and spalt (sal), as well as the unpaired (upd) and upd2 genes, which encode ligands for the Janus kinase/signal transducer and activator of transcription (JAK/STAT) pathway receptor (21).

At later stages, STAT activity feeds back directly into Abd-B expression by binding to an Abd-B enhancer and activating it. Among their functions, both Abd-B and STAT also participate in the activation of crumbs, a central cell-polarity gene involved in posterior spiracle formation, thereby promoting spiracle organogenesis (21).

Thus, the study demonstrates a positive-feedback loop between the JAK/STAT signaling pathway and Abd-B, together with their convergence on downstream genes that drive posterior spiracle organogenesis.

## LIM-homeobox 2/LIM-homeobox 9 (LHX2/LHX9) and the fibroblast growth factor 10–fibroblast growth factor 8 (FGF10–FGF8) feedback loop in limb development

A well-established example of reciprocal signaling between adjacent tissues is found in the developing vertebrate limb bud. The apical ectodermal ridge, or AER, is a specialized epithelial structure located at the distal edge of the limb bud. Beneath it lies the distal limb mesenchyme, classically referred to as the progress zone, which contains proliferative, undifferentiated progenitor cells that contribute to patterned limb outgrowth.

During early limb development, mesenchymal cells in the distal limb bud produce FGF10. FGF10 signals to the overlying ectoderm and promotes the formation and maintenance of the AER. Once formed, the AER produces fibroblast growth factors, especially FGF8, and in some contexts also FGF4 and FGF2. These AER-derived FGFs signal back to the underlying mesenchyme, maintaining its proliferation and delaying differentiation (22–24).

As limb bud growth proceeds, some mesenchymal cells are gradually displaced away from the AER. Once they leave the influence of AER-derived signals, they begin to differentiate into cartilage, bone, and other connective tissues, thereby contributing to proximal-to-distal limb patterning.

The reciprocal signaling loop can be represented schematically as follows:

FGF10 in distal limb mesenchyme → FGF8 in the AER → maintenance of FGF10 in distal limb mesenchyme

The LIM-homeobox transcription factors LHX2 and LHX9 are expressed in the limb mesenchyme, particularly in the distal mesenchymal region subjacent to the AER. They are therefore positioned in the same cellular compartment that receives AER-derived FGF signals and expresses FGF10.

One study examined the possible role of LHX2 and LHX9 in maintaining FGF10 expression. Loss-of-function experiments showed that LHX2 and LHX9, together with their cofactor LIM domain-binding protein 1 (LDB1), are required for proper limb outgrowth and for the maintenance of FGF10 expression after limb bud initiation. In the mutants, FGF10 expression is reduced in the distal mesenchyme, followed by a reduction in FGF8 expression in the AER. This indicates disruption of the reciprocal FGF10–FGF8 loop (25).

A more recent study examined the upstream regulation of LHX2 by signaling pathways in the distal limb mesenchyme. It identified two cis-regulatory modules (CRMs) within the LHX2 locus that contain binding sites for ETS transcription factors and T-cell factor/lymphoid enhancer factor (TCF/LEF) transcription factors (26). ETS transcription factors are associated with FGF/extracellular signal-regulated kinase (ERK) signaling, whereas TCF/LEF transcription factors are associated with Wnt/β-catenin signaling. The study therefore suggested that FGF8 and Wnt signaling pathways participate in the regulation of LHX2 expression in the distal limb mesenchyme.

Together, these findings suggest a positive-feedback circuit in which AER-derived FGF8 signaling contributes to LHX2 expression in the distal mesenchyme, while LHX2/LHX9/LDB1 activity helps maintain FGF10 expression in the same mesenchymal cells. FGF10 then acts back on the AER to sustain FGF8 expression. This creates an inter-tissue-level feedback loop linking signaling centers, transcription factors, and reciprocal ligand production.

## Zinc finger E-box-binding homeobox 1 (ZEB1) at the center of EMT in cancer and signaling pathway positive feedback

In different cancer types, driver mutations activate several major pathways, including RTK–RAS signaling, TP53, the gene encoding p53, and genes related to the maintenance of epithelial identity, such as adenomatous polyposis coli (APC) and CDH1, the gene encoding E-cadherin.

Because this work focuses on probable positive-feedback loops linking RTK–RAS signaling to homeobox genes, TP53 is omitted from these schemes. Although TP53 mutations are among the most frequent alterations in cancer and may affect the expression of various transcription factors, including homeobox genes, p53-dependent pathways are not included here.

ZEB1 is a zinc finger E-box-binding homeobox transcription factor and one of the central regulators of epithelial–mesenchymal transition, or EMT. It is the homeobox gene placed at the center of this paragraph. In the proposed scheme, ZEB1 is positioned at the convergence of two signaling arms that can cooperate in promoting an EMT-associated cellular state (10).

The first arm can be defined as the RAS–ERK arm. Activating mutations in RAS, or in upstream RTKs or RAF, maintain ERK signaling in an active state. Active ERK regulates downstream transcription factors, including ETS family members and AP-1. ETS factors, particularly ETS1, have been shown to activate the ZEB1 promoter and induce ZEB1 expression (27, 28).

Increased ZEB1 expression then promotes an EMT-associated transcriptional state and may reinforce signaling responsiveness by altering receptor programs, including the FGF/fibroblast growth factor receptor (FGFR) axis. This occurs through increased FGFR expression and the production of active spliced variants in various cancer cells (29–31).

This forward-reinforcing circuit, promoted by FGFR activity, may strengthen the RAS–ERK pathway and further support the EMT-associated cellular state.

The second arm can be named the β-catenin arm. Mutations in either APC or CDH1 can increase β-catenin availability, although by different mechanisms. CDH1 encodes E-cadherin, which anchors β-catenin at adherens junctions at the plasma membrane. Loss of E-cadherin therefore weakens epithelial adhesion and may release junction-associated β-catenin (32).

APC, by contrast, is a core component of the cytoplasmic β-catenin destruction complex, together with Axin, glycogen synthase kinase 3β (GSK3β), and casein kinase 1 (CK1). This complex normally phosphorylates β-catenin and targets it for degradation. APC loss impairs β-catenin turnover, allowing β-catenin to accumulate in the cytoplasm and translocate to the nucleus (33).

In the nucleus, β-catenin cooperates with TCF/LEF transcription factors and can directly activate ZEB1 transcription (34).

Increased ZEB1 then represses E-cadherin expression, further weakening epithelial adhesion and potentially reinforcing β-catenin availability and EMT-associated invasiveness (32).

Cross-talk from the RAS–ERK pathway may further support this arm by enhancing β-catenin signaling output through phosphorylation-dependent mechanisms that increase β-catenin stability, nuclear activity, or transcriptional activity (35).

The positive-feedback loops proposed here are reconstructed from separate lines of evidence rather than proven as complete circuits in a single experimental system. Nevertheless, their individual components converge on a coherent biological sequence: APC loss activates β-catenin signaling, oncogenic KRAS strengthens MAPK activity, and both pathways promote EMT-associated programs in which ZEB1 plays a central role. The resulting loss of epithelial adhesion, altered receptor signaling, invasive behavior, and stabilization of a mesenchymal state, all of which can also be promoted by ZEB1, support the broader principle that ZEB1 can participate in self-reinforcing mechanisms that sustain EMT and tumor plasticity.

Two additional components can be added to this scheme: transforming growth factor-β (TGF-β) signaling and cell division control protein 42 homolog (CDC42).

In normal epithelial cells, TGF-β signaling through SMAD2/3 often acts as a tumor-suppressive pathway by limiting proliferation and maintaining tissue homeostasis. In later stages of cancer progression, however, cancer cells may become resistant to the growth-inhibitory effects of TGF-β, allowing the pathway to be redirected toward EMT, invasion, metastasis, and therapy resistance. In cancer cells, TGF-β can activate ZEB1 expression through SMAD-dependent and SMAD-independent mechanisms. Once ZEB1 rises, it represses the microRNA-200 (miR-200) family. This is important because miR-200 normally suppresses ZEB1 and ZEB2. Thus, when ZEB1 represses miR-200, it removes one of the main brakes on its own expression. In cooperation with RTK–RAS signaling and other oncogenic pathways, this network can sustain high ZEB1 expression and stabilize EMT-associated traits (36–38).

**Figure 1:**
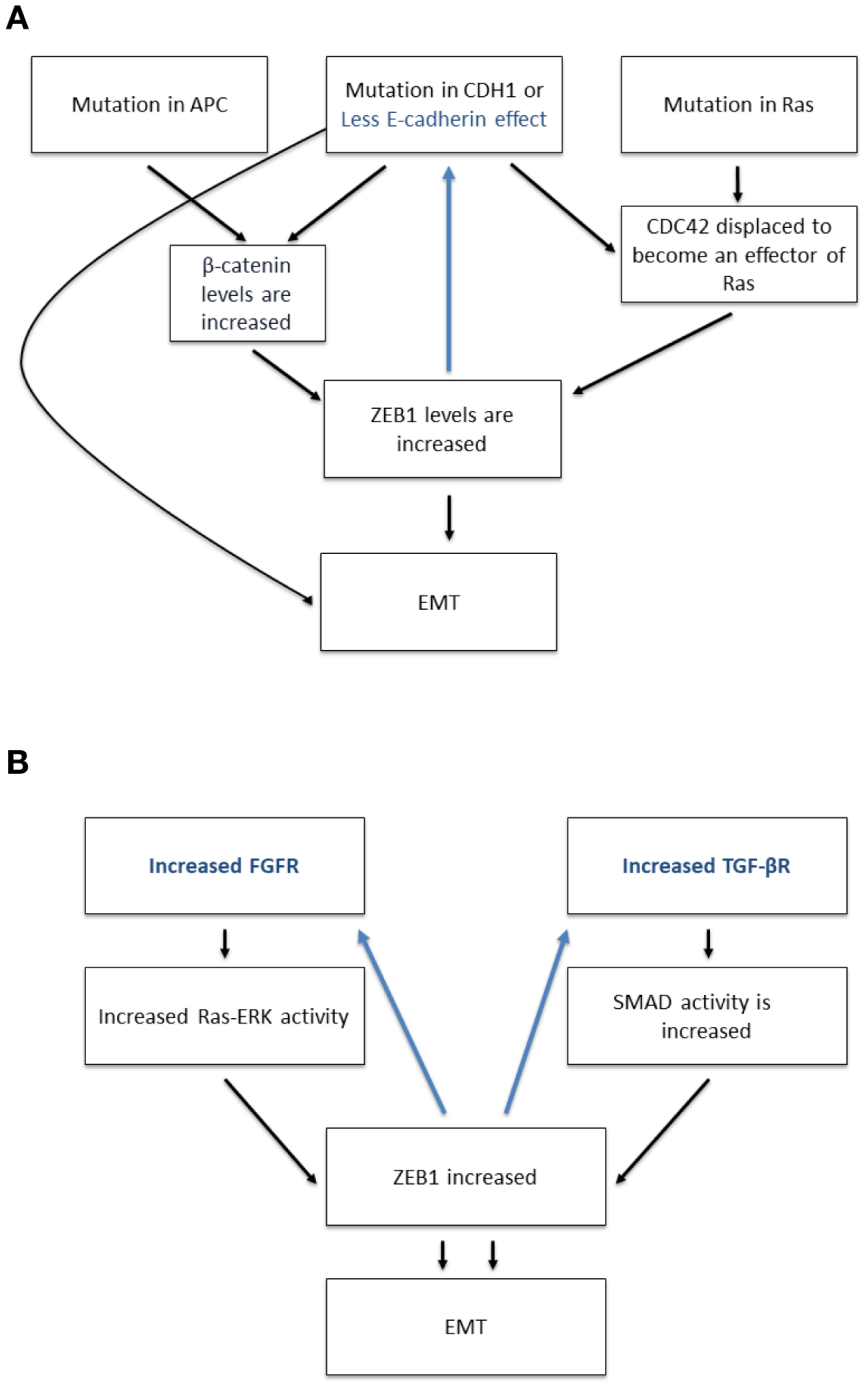
**(A)** Early mutations in the β-catenin arm and the RAS–ERK arm initiate ZEB1-related positive feedback and the resulting EMT. This figure illustrates how mutations in either APC or CDH1 activate β-catenin-dependent ZEB1 expression. It also shows how RAS mutation activates the RAS–ERK arm of ZEB1 expression. The blue arrows and blue text indicate the ZEB1 positive-feedback effect. The role of CDC42 in these early steps is also shown. **(B)** Later stages of ZEB1 activation result from the ZEB1 positive-feedback effect. This figure illustrates the reinforcement of ZEB1 activity and the further induction of EMT. The blue arrows and blue text reflect the fact that this figure describes the consequences of an already active ZEB1 state, including both reinforcement of the RAS–ERK pathway through the activation of a new RTK, FGFR, and reinforcement of the TGF-β pathway.

CDC42 provides an additional connection between epithelial adhesion, polarity, and signaling plasticity. CDC42 regulates apical junction formation and epithelial polarity, while E-cadherin-mediated cell-cell adhesion can itself activate CDC42, indicating a close functional relationship between adherens junctions and CDC42 activity. In parallel, CDC42 can also act downstream of receptor tyrosine kinase signaling, as shown for hepatocyte growth factor/MET (HGF/MET) signaling in epithelial cells, and genetic studies indicate that it is required for the full transforming activity of oncogenic RAS. Together, these findings position CDC42 as a point of convergence between epithelial adhesion, polarity regulation, RTK signaling, and RAS-driven transformation (39–41). **Figures 1A, B** describe some of the main genes and chains of interaction responsible for ZEB1 activation and for the positive-feedback loops it coordinates, as discussed in this chapter.

## HOXB7–epidermal growth factor receptor/human epidermal growth factor receptor 2 (EGFR/HER2) positive feedback in endocrine-resistant breast cancer

The following chapter describes one of the most complete examples of a positive-feedback loop involving a homeobox gene in cancer. This loop was characterized across two published studies.

The first study showed that HOXB7 overexpression in estrogen receptor (ER)-positive breast cancer cells confers tamoxifen resistance through increased EGFR expression and signaling. The authors suggested that elevated HOXB7 expression is one of the key steps in the acquisition and maintenance of resistance to endocrine therapy in ER-positive breast cancer. Accordingly, HOXB7 was proposed to serve as a critical regulator in the transition of breast cancer cells toward estrogen independence, tamoxifen resistance, and a more aggressive phenotype (42).

A second study further investigated the interaction between HOXB7 and growth-factor signaling and delineated a more complete positive-feedback loop. The study showed that HOXB7 interacts with estrogen receptor α (ERα) at the promoters of many ERα-target genes and enhances the expression of several ER-regulated genes, including HER2 and MYC. Using several complementary strategies, the authors demonstrated that MYC protein is stabilized through phosphorylation mediated by EGFR–HER2 signaling, and that this stabilization correlates with HOXB7 levels. Stabilized MYC suppresses miR-196a, a microRNA that represses HOXB7. This suppression relieves miR-196a-mediated inhibition of HOXB7, leading to increased HOXB7 expression and transcriptional activity (11).

The loop can therefore be summarized as shown below.

HOXB7 interaction with ERα → increased HER2 and MYC expression → EGFR/HER2 signaling → MYC stabilization → repression of miR-196a → increased HOXB7 expression

An important point, not fully captured in the short scheme, is that increased MYC expression itself is one of the transcriptional outcomes of HOXB7 interaction with ERα at ER-regulated target genes. Thus, MYC participates in the loop at two levels, first as a transcriptional target induced by the HOXB7–ERα complex, and then as a stabilized protein downstream of EGFR/HER2 signaling that represses miR-196a and thereby reinforces HOXB7 expression.

## Lessons for identifying positive-feedback loops in signaling pathways

Almost all the examples discussed in this Perspective contain a multistep puzzle, in which separate pieces, when connected together, form the larger picture of signaling-pathway positive feedback. In this way, the “detective” work involved in identifying these loops becomes an invitation to prove them in further functional studies. Some positive-feedback loops in this Perspective have already been described as such by the original authors.

This Perspective tries to emphasize the centrality of positive-feedback loops in normal cellular function and cancer formation, since only when such centrality is recognized can these mechanisms be actively searched for, rather than discovered only incidentally.

Identifying the precise mechanism by which a positive-feedback loop operates may be difficult, especially when trying to determine how signaling pathways activate homeobox genes. Examples such as the breast cancer loop described above, in which suppression of the suppressor miR-196a leads to increased HOXB7 expression, should therefore be taken into consideration (11). As the role of miRNAs in gene regulation becomes increasingly central, their involvement may change both experimental results and their interpretation (11, 36). miRNAs do not necessarily change the transcription rate of the gene being analyzed, and a loop that contains miRNA suppression does not necessarily involve direct binding of a transcription factor to an enhancer or promoter region of the gene under inquiry.

Thus, initially, a positive-feedback loop should be suspected whenever there is an association between two genes, one encoding a receptor and the other encoding a transcription factor.

As illustrated by the HOXB7 example, the association of functionally active RTK signaling with high expression of specific homeobox genes in the same tumor may serve as an initial hint for such a loop (42).

After such an association is found, functional studies can be designed to test possible interactions in both directions. First, they can examine whether transcription factors activated by the signaling pathway bind enhancers or promoters of homeobox genes. Second, they can examine whether homeobox transcription factors regulate enhancers or promoters of receptors, ligands, or other signaling components active in the same cellular context.

Mechanisms such as reduced miRNA-mediated repression leading to increased homeobox gene activity, together with the upstream events responsible for that reduction, should be actively sought.

## From concept to therapy: disrupting homeobox-driven signaling reinforcement

The working hypothesis proposed here is that, during cancer progression, the homeobox gene transcriptome may coordinate positive-feedback loops within signaling pathways and thereby support tumor plasticity. If so, altering this transcriptome may disrupt both the self-reinforcing signaling state and the plasticity it supports.

Because homeobox genes are largely regarded as undruggable, modulation of their expression patterns, including reduction of their expression, may be achieved through the use of epigenetic drugs. Homeobox genes are highly responsive to epigenetic regulation, and their expression patterns can change in response to different classes of epigenetic agents.

Thus, a therapeutic combination that includes an RTK- or RAS-blocking agent, selected according to the mutation present in the tumor cell, together with an effective epigenetic drug, may interfere with this self-reinforcing loop. Such combinations may also function as a form of vertical signaling-pathway inhibition, targeting both the upstream signaling input and the downstream transcriptional state.

Increasing evidence from *in vitro* and animal models has demonstrated encouraging results with combinations that include MAPK or RAS inhibitors together with epigenetic drugs, particularly histone deacetylase (HDAC), enhancer of zeste homolog 2 (EZH2), and bromodomain and extraterminal (BET) inhibitors (43–45). Still, these strategies have so far yielded only limited therapeutic benefit in clinical trials involving common, clinically important cancers (46).

One notable example is the triple combination of azacitidine, venetoclax, and gilteritinib, which resulted in high response rates in patients with newly diagnosed or relapsed FMS-like tyrosine kinase 3 (FLT3)-mutated acute myeloid leukemia (47).

The identification of effective drug combinations should begin with systematic screening in cancer cell lines bearing RTK or RAS mutations. These cell lines should be treated with the appropriate RTK- or RAS-blocking agent together with different classes of epigenetic drugs. The response should be assessed both by the extent of cell killing and by changes in homeobox gene expression, preferably including reduced expression of homeobox genes associated with the malignant cellular state after treatment.

## Data Availability

The original contributions presented in the study are included in the article/supplementary material. Further inquiries can be directed to the corresponding author.
